# SG-APSIC1086: Case series: Examining healthcare-associated infection cases caused by *Candida auris* at Cho Ray Hospital, Vietnam

**DOI:** 10.1017/ash.2023.56

**Published:** 2023-03-16

**Authors:** Thoa Vo Thi Hong, Phung Manh Thang, Tran Thi Thu Ha, Nguyen To Nhu, Bui Chi, Amber Vasquez

**Affiliations:** Cho Ray Hospital, Ho Chi Minh City, Vietnam; Cho Ray Hospital, Ho Chi Minh City, Vietnam; Program For Appropriate Technology in Health, Hanoi, Vietnam; Program For Appropriate Technology in Health, Hanoi, Vietnam; Program For Appropriate Technology in Health, Hanoi, Vietnam; Program For Appropriate Technology in Health, Hanoi, Vietnam

## Abstract

**Objectives:** *Candida auris* was first detected in Japan in 2009 and has been reported in >47 countries, typically causing outbreaks in healthcare settings. According to the US Centers for Disease Control and Prevention, this pathogen causes death in more than one-third of infected patients. This study describes characteristics of healthcare-associated infections (HAIs) related to *C. auris* and infection prevention and control (IPC) measures applied to control transmission in Cho Ray Hospital, a tertiary-care, referral, general hospital in southern Vietnam. **Methods:** We reviewed medical records of all patients with HAIs caused by *C. auris* at Cho Ray Hospital between April 2020 and March 2021, as well as the IPC measures applied for these patients. **Results:** Overall, 5 HAI cases caused by *C. auris* were identified in 5 patients, including 2 catheter-associated urinary tract infections, 2 ventilator-associated pneumonia cases, and 1 surgical site infection. These cases were sporadically detected in 4 different clinical departments; 2 cases occurred in the respiratory department in April and August 2020. The average age of the patients was 63, and 4 of 5 patients were male. The average hospital stay was 27.2 days; 4 patients died and 1 was discharged. IPC interventions were implemented to immediately respond to *C. auris* infection cases, including isolating the patients, applying standard and transmission-based precautions, supplying adequate personal protective equipment, cleaning environment surfaces and medical equipment in the patient’s room, and marking isolation areas with signage. No additional cases of *C. auris* infection were detected in the affected units. **Conclusions:** *C. auris* can spread in healthcare settings via contact with contaminated equipment and surfaces or from person to person, causing outbreaks in hospitals and leading to severe illness and high mortality for patients. Prompt application of appropriate IPC measures effectively helped prevent additional cases of *C. auris* in our hospital.

